# Association between long-term sedentary behavior and depressive symptoms in U.S. adults

**DOI:** 10.1038/s41598-024-55898-6

**Published:** 2024-03-04

**Authors:** Yuyang Guo, Kaixin Li, Yue Zhao, Changhong Wang, Hongfei Mo, Yan Li

**Affiliations:** 1 Department of Physical Education, Zhengzhou Railway Vocational and Technical College, Zhengzhou, Henan People’s Republic of China; 2 Zhengzhou University, Zhengzhou, Henan People’s Republic of China; 3 Synergetic Innovation Center of Kinesis and Health, School of Physical Education (Main Campus), Zhengzhou University, Zhengzhou, Henan People’s Republic of China; 4 The Second Affiliated Hospital of Xinxiang Medical University, Xinxiang, Henan People’s Republic of China; 5 The Second Affiliated Hospital of Zhengzhou University, Zhengzhou, Henan People’s Republic of China

**Keywords:** Depressive symptoms, Long-term sedentary behavior, Moderate-to-sever depressive symptoms, NHANES, Physical activity, Health care, Medical research, Risk factors

## Abstract

The study aimed to investigate the association between long-term sedentary behavior (LTSB) and depressive symptoms within a representative sample of the U.S. adult population. Data from NHANES 2017–2018 were used, encompassing information on demographics, depressive symptoms, physical activity (PA), and LTSB. Depressive symptoms were identified using the Patient Health Questionnaire (PHQ-9), with “depressive symptoms” defined as a PHQ-9 score of ≥ 5, and “moderate to severe depressive symptoms (MSDS)” defined as a PHQ-9 score of ≥ 10. PA and LTSB were assessed through the Global Physical Activity Questionnaire, where LTSB was interpreted as sedentary time ≥ 600 min. Restricted Cubic Spline (RCS) curves were utilized to observe potential nonlinear relationships. Binary Logistic regressions were conducted to analyze the associations. A total of 4728 participants (mean age 51.00 ± 17.49 years, 2310 males and 2418 females) were included in the study. Among these individuals, 1194 (25.25%) displayed depressive symptoms, with 417 (8.82%) exhibiting MSDS. RCS curves displayed increased risk of depressive symptoms with prolonged sedentary duration. Logistic regression models indicated significant associations between LTSB and depressive symptoms (OR 1.398, 95% CI 1.098–1.780), and LTSB and MSDS (OR 1.567, 95% CI 1.125–2.183), after adjusting for covariates. These findings suggest that LTSB may act as a potential risk factor for both depressive symptoms and MSDS in the studied population.

## Introduction

Mental disorders are the major causes of the global health-related burden, with depressive symptoms being the primary contributor to this burden^1^, which severely affects quality of life^2^. Depression is also the leading cause of disability worldwide, affecting approximately 280 million people and causing more than 47 million disabilities yearly^3^. Hasin predicted that the 12-month and lifetime prevalence of depression was 10.4% and 20.6% in Americans^4^. However, recent surveys suggest that the incidence of depressive symptoms in U.S. adults is increasing^5,6^. Also with the outbreak of COVID-19 pandemic in recent years, risk factors such as economic stress and social isolation have also briefly led to an increase in depressive symptoms^7^. Given the high prevalence of depressive symptoms and its significant impact on quality of life, it is imperative to identify the risk factors that contribute to its development and implement effective intervention measures.

Physical activity (PA) was previously argued to be strongly associated with depressive symptoms^8–10^. Exercise therapy was shown to have a similar effect as drug therapies for major depression^11^. PAs are defined as activities that require a metabolic equivalent of more than 1.5 Metabolic Equivalents (METs) during waking hours, while sedentary behavior generally refers to activities that require a metabolic equivalent of less than 1.5 METs^12,13^. The decrease of PA inevitably lead to the increase of sedentary behavior. World Health Organization suggested that adults aged 18–64 should limit the amount of sedentary time and replace them with aerobic activity to prevent chronic conditions^14^. The duration of “10 h” has been widely acknowledged as a critical threshold for Long-term sedentary behavior (LTSB)^15–17^. Prolonged sedentary behavior exceeding 10 h can have detrimental effects on human health. A comprehensive review by authoritative sources highlighted that engaging in LTSB significantly increases the risk of chronic diseases and all-cause mortality^18^. In addition, studies have shown that the negative effect of LTSB was not always offset by the benefits of PA^19^, and LTSB may be an independent risk factor for some chronic conditions. Therefore, it has started to recognize the impact of LTSB as an independent risk factor for depressive symptoms.

Previous studies demonstrated that LTSB may lead to emotional disorders in university students^20^, adolescents^21,22^ and senior citizens^23^. However, these studies lacked a unified description of the U.S. population, sample size and sample distribution characteristics were generally limited, and the representativeness was also weak. Therefore, a reliable large sample cross-sectional survey in a representative sample of the U.S. adult population is particularly essential.

The aim of this study is to explore the association between LTSB and depressive symptoms through rigorous analysis and evaluation with the samples from a larger population.

## Materials and methods

### Study population

This study was a cross-sectional survey that followed the STROBE checklist. Participants were sourced from the National Health and Nutrition Examination Survey (NHANES), a population-based cross-sectional survey designed to collect information about the health and nutrition situation of the U.S. household population. A stratified multistage sampling design was used to obtain a representative sample of U.S. residents aged two months and older. The NHANES protocol was approved by the National Center for Health Statistic's esearch ethics review board; all adult participants provided written notice of consent^24^. And the use of NHANES data as a secondary data source was also approved^25^. The present study extracted and aggregated data on demographic characteristics, depressive symptoms, PA and LTSB from the NHANES 2017–2018, and the current sample is restricted to adults aged 20 and older.

### Depressive symptoms assessment

The Patient Health Questionnaire (PHQ-9), a nine-item depression screening instrument, was used to assess the frequency of depression symptoms in the sample over the past 2 weeks. The questions were asked at the Mobile Examination Center (MEC) by trained interviewers using the Computer-Assisted Personal Interview (CAPI) system, which is programmed with built-in consistency checks to reduce data entry errors as part of the MEC interview. For each item, points ranging from 0 to 3, are associated with the response categories "not at all", "several days", "more than half the days", and "nearly every day"^26,27^. A total score ranging from 0 to 27 can be computed for participants who provide complete responses to the symptom questions. Cut-off points of 5, 10, and 20 are typically used to indicate levels of symptoms severity^27^. A PHQ-9 score of ≥ 5 is commonly considered indicative of "depressive symptoms" in general^28,29^. On the other hand, a PHQ-9 score of ≥ 10 is recognized as the threshold for "moderate-to-severe depressive symptoms (MSDS)" and serves as a clinical criterion to screen for major depression, indicating the need for intervention^30^. In this study, we categorized PHQ-9 scores into two criteria: PHQ-9 score ≥ 5 and ≥ 10 to examine the associations between LTSB and "depressive symptoms", and LTSB and "MASD", respectively. Furthermore, the last question of PHQ-9: “Over the last 2 weeks, how often have you been bothered by the following problem: Thoughts that you would be better off dead or of hurting yourself in some way?”, was used to evaluate participants' self-injury tendencies^31^.

### LTSB assessment

Participants’ sedentary duration was assessed through a respondent-level interview using the Global Physical Activity Questionnaire (GPAQ)^32^. The interview was also established in MEC by trained interviewers using the CAPI system. Sedentary duration was assessed through one question: “The following question is about sitting at school, at home, getting to and from places, or with friends, including time spent sitting at a desk, traveling in a car or bus, reading, playing cards, watching television, or using a computer. Do not include time spent sleeping. How much time do you usually spend sitting on a typical day?” The reported sedentary duration was recorded in minutes. For the purpose of this study, sedentary duration > 600 min was categorized as LTSB.

### Covariates

The covariates considered in this study include gender, age, race, education, marital status, smoking status, military service, self-injury tendency, body mass index (BMI) status, and PA. These variables were chosen based on their potential associations with specific health risks and experiences, substance use, negative health consequences of obesity, and positive health outcomes resulting from good behavior^33–35^. We have included these covariates as they may impact the primary outcome of our study. Age was divided into three groups: < 40, 40–60, and > 60. Race was divided into five groups: Hispanic, Non-Hispanic white, Non-Hispanic black, Non-Hispanic Asian, and Other. Education was divided into four groups: < 12th grade, high school, some college, college and above. Marital status was classed as married and not married (living with a partner, widowed, divorced, separated, never married). Smoking status was classed as smoking (smoked 100 cigarettes in life) and non-smoking (did not smoke 100 cigarettes in life)^36^. BMI was measured by trained technicians using standardized equipment during MEC physical examination, and BMI status was categorized into three categories: Underweight (≤ 18.9 kg/m^2^), Normal weight (19.0–29.9 kg/m^2^) and Obese (≥ 30.0 kg/m^2^)^37^. Considering that different types of PAs affect depression in different directions, we selected two physical activity covariates. The first, work physical activity (OPA), a type of physical activity that promotes the development of depressive symptoms^38^, and the second, leisure-time physical activity (LTPA), a type of physical activity pair that inhibits the development of depressive symptoms^39^. Both OPA and LTPA were assessed by the GPAQ and classified into three levels: inactive, moderate and vigorous^40^.

### Statistical analyses

Initially, we aggregated the extracted information, excluded missing and missing or irrelevant data, utilizing Microsoft Excel 2010. Adults aged 20 years and older from the NHANES 2017–18 cycle with complete information on independent variable (LTSB) and dependent variable (depressive symptoms) were included in the analyses.

Subsequently, we employed SPSS 26.0 for conducting descriptive statistics, inter-group comparisons, and binary logistic regression analysis. In the inter-group analysis, we categorized the dependent variables into "depressive group" and "non-depressive group", and assess the variances in independent variable and covariates between the two groups. Categorical variables were evaluated using the chi-square test, while continuous variables were analyzed using the rank-sum test. Logistic regression was carried out to analyze associations between LTSB and depressive symptoms, as well as LTSB and MSDS. Covariates that were statistically significant in the inter-group comparisons were included in the regression models. In order to eliminate the interference of covariates, we have established the following models: Model I: Primary model, without adjusting for any covariates. Model II: Adjusted for the independent variable in Model I plus demographic covariates (gender, race, marital status). Model III: Adjusted the variables in Model II all covariates (plus smoking, LTPA, BMI).

Concurrently, we utilized the R software to generate Restricted Cubic Spline (RCS) curves. The RCS curves were created for "LTSB-Depressive Symptoms" and "LTSB-MSDS," with the dependent variable serving as the horizontal axis, and the odds ratio (OR) along with the 95% confidence interval (CI) representing the vertical axis. This visualization allowed us to investigate potential nonlinearity within these two associations. P-values of less than 0.05 were considered statistically significant (2-sided tests).

### Ethics approval and consent to participate

All procedures performed in the study were in accordance with the Declaration of Helsinki. The study protocols for NHANES were approved by the National Center for Health Statistics (NCHS) Research Ethics Review Board (Protocol#2017–1). All adult participants provided written notification of consent before participating in the study.

## Results

### Demographic characteristics

Out of the 5569 participants initially included in the study, 1291 individuals (23.18%) had missing data for LTSB or depression were excluded. Consequently, a total of 4728 adults aged 20 years or older, who participated in the NHANES cycle 2017–2018, were included in the final analysis. The participants had a mean age of 51.00 ± 17.49 years at the time of examination, comprising 2310 males and 2418 females, with an average sedentary duration of 330.61 ± 119.67 min. Significant statistical differences were observed in gender (*P* < 0.001), age (*P* = 0.021), race (*P* = 0.043), marital status (*P* < 0.001), smoking status (*P* < 0.001), sedentary duration (*P* = 0.038), LTSB (*P* = 0.001), BMI (*P* < 0.001), self injury tendency (*P* < 0.001), OPA (*P* = 0.007) and LTPA (*P* < 0.001) between the depressive group and the non-depressive group. However, there were no statistically significant differences in military service (*P* = 0.335) and education (*P* = 0.068) between the two groups (See Table 1).

**Table 1:** Sample demographic characteristics, by depressive symptoms.

Characteristics	Sample capacity (%)N = 4728		Non-depressive group n = 3534 (73.4%)		Depressive group n = 1194 (26.6%)		*P* value
Gender							< 0.001
Male	2310	(48.86)	1826	(51.67)	484	(32.44)	
Female	2418	(51.14)	1708	(48.33)	710	(59.46)	
Age group							0.021
< 40	1443	(30.52)	1092	(30.90)	351	(29.40)	
40–60	1646	(34.81)	1191	(33.70)	455	(38.11)	
> 60	1639	(34.67)	1251	(35.40)	388	(52.50)	
Race							0.043
Hispanic	1060	(22.42)	780	(22.07)	280	(23.45)	
Non-Hispanic White	1675	(35.43)	1198	(33.90)	477	(39.95)	
Non-Hispanic Black	1112	(23.52)	843	(23.85)	269	(22.53)	
Non-Hispanic Asian	644	(13.62)	555	(15.70)	89	(7.45)	
Other	237	(5.01)	158	(4.47)	79	(6.62)	
Military service							0.335
Yes	482	(10.19)	369	(10.44)	113	(9.46)	
No	4246	(89.81)	3165	(89.56)	1081	(90.54)	
Education							0.068
< 12th grade	871	(18.42)	596	(16.86)	275	(23.03)	
High school	1145	(24.22)	830	(23.49)	315	(26.38)	
Some college	1552	(32.83)	1128	(31.92)	424	(35.51)	
College and above	1160	(24.53)	980	(27.73)	180	(15.08)	
Marital status							< 0.001
Married	2377	(50.27)	1920	(54.33)	457	(38.27)	
Not married	2351	(49.73)	1614	(45.67)	737	(61.73)	
Smoking status							< 0.001
Smoking	2009	(42.49)	1381	(39.08)	628	(52.60)	
Non-smoking	2719	(57.51)	2153	(60.92)	566	(47.40)	
Sedentary duration (min)	330.61 ± 119.67		328.51 ± 196.83		352.33 ± 226.13		0.038
LTSB							0.001
> 600 min	355	(7.51)	239	(6.76)	116	(9.72)	
≤ 600 min	4373	(92.49)	3295	(93.24)	1078	(90.28)	
BMI status							< 0.001
Underweight	104	(2.20)	69	(1.95)	35	(2.93)	
Normal weight	2610	(55.20)	2067	(58.49)	543	(45.48)	
Obese	2014	(42.60)	1398	(39.56)	516	(51.59)	
Self-injury tendency							< 0.001
Yes	173	(3.66)	13	(0.40)	160	(13.40)	
No	4555	(96.34)	3521	(99.6)	1034	(86.60)	
OPA							0.007
Inactive	2489	(52.64)	1907	(53.96)	582	(48.74)	
Moderate	1069	(22.61)	774	(21.90)	295	(24.71)	
Vigorous	1170	(24.75)	853	(24.14)	317	(26.55)	
LTPA							< 0.001
Inactive	2486	(52.58)	1719	(48.64)	767	(64.24)	
Moderate	1102	(23.31)	875	(24.76)	227	(19.01)	
Vigorous	1140	(24.11)	940	(26.60)	200	(16.75)	

### Depressive symptoms in the present study

Out of the 4278 participants surveyed, 1194 (25.25%) exhibited depressive symptoms (PHQ-9 score ≥ 5). Among the individuals with depressive symptoms, 777 (16.43%) participants demonstrated mild depressive symptoms, whereas the remaining 417 (8.82%) participants exhibited MSDS (PHQ-9 score ≥ 10), which were generally believed requiring interventions.

### RCS curves of sedentary duration and depressive symptoms

**Figure 1:**
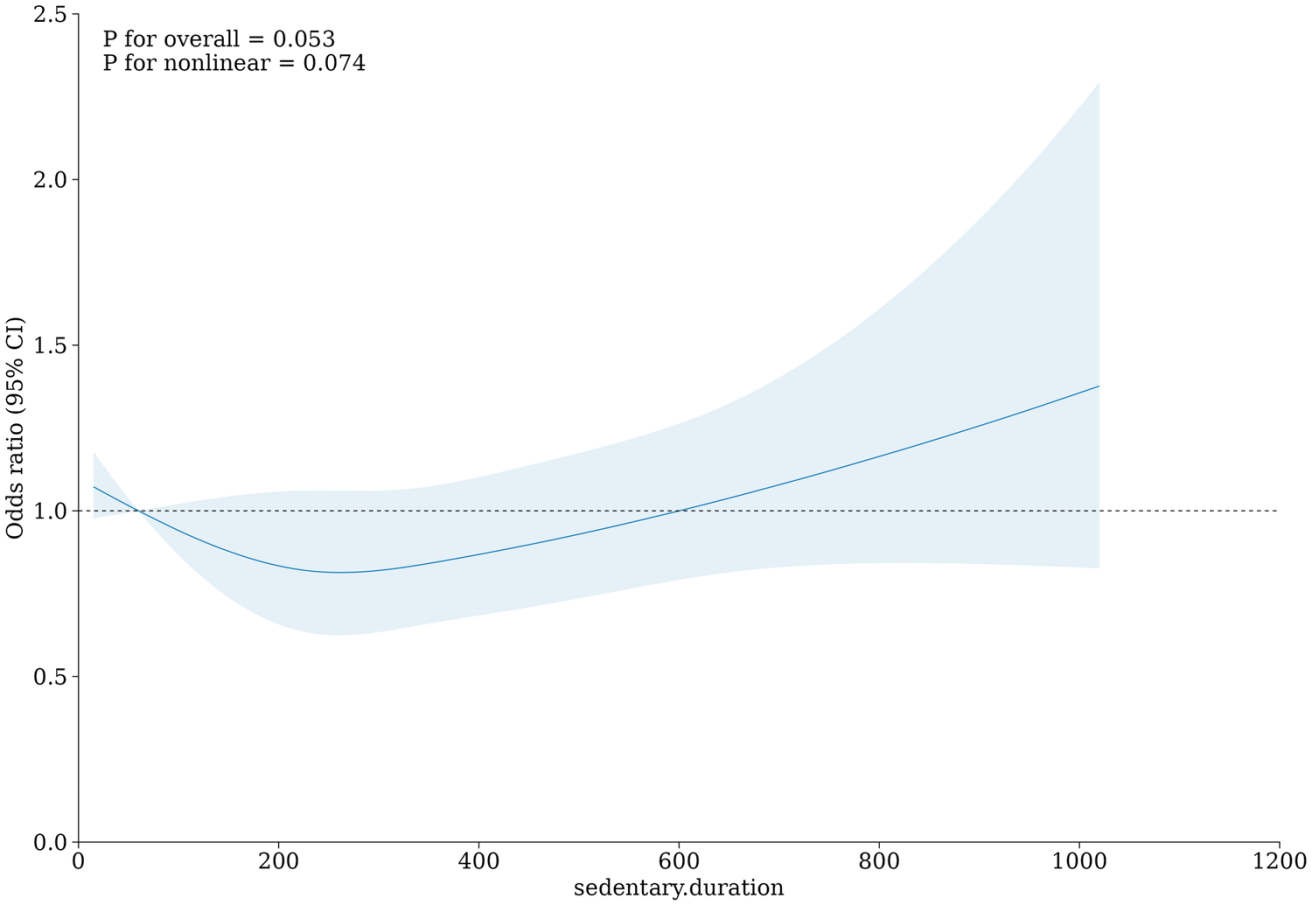
RCS curve of association between sedentary duration (min) and depressive symptoms.

**Figure 2:**
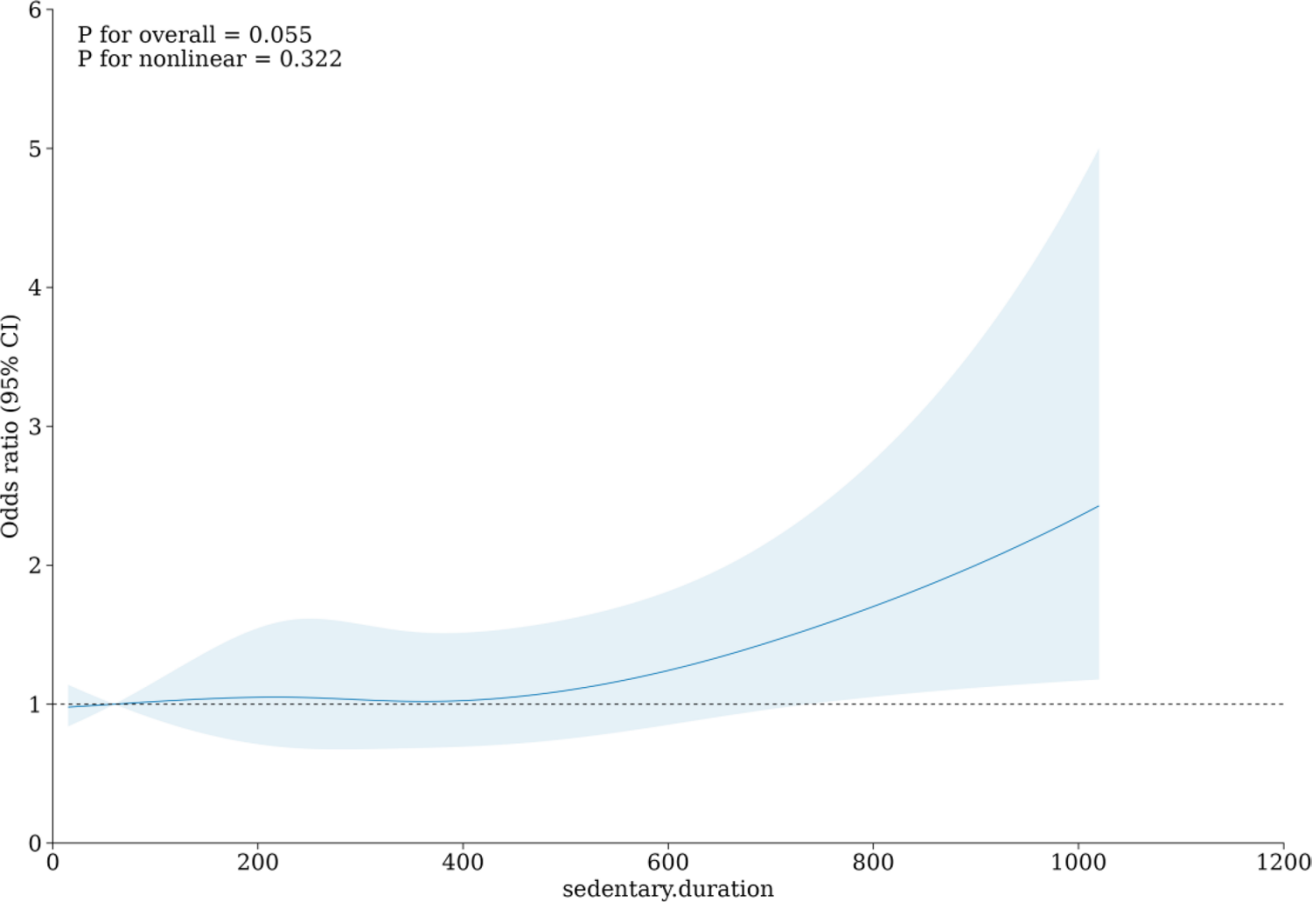
RCS curve of association between sedentary duration (min) and MSDS.

The results revealed that the mean odds ratio (OR) for the association between sedentary duration and depressive symptoms remained below 1 until reaching 600 min, and subsequently exceeded 1 for duration exceeding 600 min. Moreover, the OR increased with longer sedentary duration (see Fig. 1). In the case of the association between sedentary duration and MSDS, the mean OR consistently exceeded 1 and exhibited an upward trend with longer sedentary duration (see Fig. 2).

### Logistic regression analyses

In the analysis of the association between LTSB and depressive symptoms, our results revealed a significant positive association. Before adjusting for covariates, participants engaging in LTSB were found to have higher odds of experiencing depressive symptoms (OR 1.484, 95% CI 1.176–1.817). This association remained significant after adjusting for covariates (OR 1.398, 95% CI 1.098–1.780). Specifically, individuals who engaged in sedentary behavior for more than 600 min per day had a 39.8% higher likelihood of developing depressive symptoms (See Table 2).

**Table 2:** Association between LTSB and depressive symptoms.

Mode	*b*	S.E	*Wald*	*P* value	OR (95% CI)
Mode I^a^	0.394	0.118	11.084	0.001	1.484 (1.176–1.817)
Mode II^b^	0.426	0.121	12.414	< 0.001	1.531 (1.208–1.939)
Mode III^c^	0.335	0.123	7.377	0.007	1.398 (1.098–1.780)

^a^Primary model, no adjusting for any covariates.

^b^Adjusted for the independent variable in Model I plus socio-demographic covariates.

^c^Adjusted for all covariates.

In the analysis of the association between LTSB and MSDS, our results also revealed a significant positive association. Before adjusting for covariates, participants engaging in LTSB were found to have higher odds of experiencing MSDS (OR 1.697, 95% CI 1.229–1.342). This association remained significant after adjusting for covariates (OR 1.567, 95% CI 1.125–2.183). The results suggest that individuals with LTSB had a rather higher likelihood (56.7%) to develop MSDS (See Table 3).

**Table 3:** Association between LTSB and MSDS.

Mode	*b*	S.E	*Wald*	*P* value	OR (95% CI)
Mode I^a^	0.529	0.164	10.330	0.001	1.697 (1.229–1.342)
Mode II^b^	0.546	0.166	10.787	0.001	1.727 (1.246–2.393)
Mode III^c^	0.449	0.169	7.047	0.008	1.567 (1.125–2.183)

^a^Primary model, no adjusting for any covariates.

^b^Adjusted for the independent variable in Model I plus socio-demographic covariates.

^c^Adjusted for all covariates.

## Discussion

### LTSB and depressive symptoms

This study identified a direct association between prolonged sedentary duration and an increased risk of depressive symptoms, indicating that LTSB serves as a potential risk factor for depressive symptoms. In following discussion, we will delve into the reasons why LTSB represents a risk factor for depression across three dimensions: physiological, psychological, and social.

From a physiological standpoint, LTSB impacts physical health in various ways, potentially elevating the risk of depression. Prolonged sitting results in reduced PA levels, impacting cardiovascular health and heightening the susceptibility to heart disease and stroke^41^. These physical conditions not only affect overall health but may also contribute to mental health issues like low mood and depression. Furthermore, sedentary habits can give rise to poor posture leading to issues such as muscle tension and neck/back pain^42^, which can further exacerbate or trigger depressive symptoms through somatic pain^43^.

On a psychological level, LTSB can heighten the risk of depression by influencing an individual’s cognitive and emotional states. Prolonged sedentary duration may induce distraction and decreased productivity^44^, leading to heightened feelings of stress and anxiety. Additionally, sedentary lifestyles can restrict PA and social interactions, diminishing feelings of enjoyment and fulfillment^45^, thereby amplifying the likelihood of depression. Furthermore, sedentary patterns may impact an individual's sleep quality^46^, with restful sleep being crucial for maintaining mental well-being.

At the societal level, LTSB is closely linked with the fast-paced lifestyle and work environments prevalent in modern society. Many occupations necessitate prolonged sitting in front of computers, promoting sedentary habits. This fast-paced lifestyle often deprives individuals of ample opportunities for PA and social engagement, consequently escalating the risk of depression^47^. Societal perceptions and attitudes towards LTSB may also play a role in mental health outcomes. Certain cultural beliefs may view sedentary behavior as a manifestation of laziness or lack of self-discipline, potentially exerting a detrimental impact on an individual's mental health.

### Stronger association between LTSB and MSDS

When analyzing the association between LTSB and depressive symptoms, we observed a stronger association between LTSB and MSDS compared to the association between LTSB and depressive symptoms in general. We attribute this heightened association to the cumulative impact and cyclical nature of LTSB on both physical and mental well-being.

Prolonged sedentary duration often result in diminished PA levels, impacting cardiovascular health, body posture, and sleep quality^48^. These physical consequences may progressively deteriorate, ultimately leading to more severe health issues, including MSDS. Additionally, LTSB can curtail social interactions and engagement in social activities, reducing feelings of pleasure and satisfaction over time. This psychological state of decline may contribute to the development of MSDS. Furthermore, extended periods of sedentarism can engender negative life patterns, such as decreased motivation and interest^49^, which can exacerbate depressive symptoms, perpetuating a detrimental cycle.

Furthermore, it is noteworthy that MSDS may be linked to more pronounced biological alterations, such as neurotransmitter imbalances and inflammatory responses^50^. Prolonged sedentary duration has the potential to exacerbate depressive symptoms by influencing these biological processes. A sedentary lifestyle may elevate inflammatory markers in the body, and mounting evidence underscores the significant relationship between inflammation and depressive symptoms^51,52^.

### Limitations

This study has several limitations that need to be acknowledged. Firstly, somatic factors such as diabetes, cancer, and physical disability, which can potentially trigger depressive symptoms, were not included as covariates in our analysis. This omission could have influenced our findings. Secondly, we did not consider the influence of medication use, particularly the use of antidepressants, which could have a significant impact on depressive symptoms. This omission may have affected the observed associations. Thirdly, the small sample size utilized in this study may introduce bias and limit the generalizability of our findings. Future studies could consider including additional covariates and analyzing larger datasets encompassing multiple NHANES cycles of data to enhance the reliability and robustness of the results.

## Conclusions

In conclusion, this study demonstrated that LTSB is an independent risk factor for depressive symptoms in U.S. adults. Sedentary duration exceeding 600 min per day was significantly associated with depressive symptoms, underscoring the role of time in the harmful effects of sedentary duration on mental health. The results indicated that LTSB had a significant impact on depressive symptoms after adjusting for covariates. Furthermore, these associations were particularly strong in cases of moderate to sever depressive symptoms.

## Data Availability

The datasets generated and/or analyzed during the current study are available in the [NHANES] repository, [NHANES Questionnaires, Datasets, and Related Documentation (cdc.gov)]. Raw data supporting the obtained results are available at the corresponding authors.
